# Costs and resource needs for primary health care in Ethiopia: evidence to inform planning and budgeting for universal health coverage

**DOI:** 10.3389/fpubh.2023.1242314

**Published:** 2023-12-19

**Authors:** Abebe Alebachew, Engida Abdella, Samuel Abera, Ermias Dessie, Tesfaye Mesele, Workie Mitiku, Rodrigo Muñoz, Marjorie Opuni, Lyubov Teplitskaya, Damian G. Walker, Colin Gilmartin

**Affiliations:** ^1^Breakthrough International Consultancy, Addis Ababa, Ethiopia; ^2^Strategic Affairs Executive Office, Ministry of Health, Addis Ababa, Ethiopia; ^3^Sistemas Integrales, Santiago, Chile; ^4^Independent Consultant, Lausanne, Switzerland; ^5^Management Sciences for Health, Arlington, VA, United States

**Keywords:** actual cost, normative cost, essential health services package, primary health care, universal health coverage, Ethiopia

## Abstract

**Introduction:**

The Government of Ethiopia (GoE) has made significant progress in expanding access to primary health care (PHC) over the past 15 years. However, achieving national PHC targets for universal health coverage will require a significant increase in PHC financing. The purpose of this study was to generate cost evidence and provide recommendations to improve PHC efficiency.

**Methods:**

We used the open access Primary Health Care Costing, Analysis, and Planning (PHC-CAP) Tool to estimate actual and normative recurrent PHC costs in nine Ethiopian regions. The findings on actual costs were based on primary data collected in 2018/19 from a sample of 20 health posts, 25 health centers, and eight primary hospitals. Three different extrapolation methods were used to estimate actual costs in the nine sampled regions. Normative costs were calculated based on standard treatment protocols (STPs), the population in need of the PHC services included in the Essential Health Services Package (EHSP) as per the targets outlined in the Health Sector Transformation Plan II (HSTP II), and the associated costs. PHC resource gaps were estimated by comparing actual cost estimates to normative costs.

**Results:**

On average, the total cost of PHC in the sampled facilities was US$ 11,532 (range: US$ 934–40,746) in health posts, US$ 254,340 (range: US$ 68,860–832,647) in health centers, and US$ 634,354 (range: US$ 505,208–970,720) in primary hospitals. The average actual PHC cost per capita in the nine sampled regions was US$ 4.7, US$ 15.0, or US$ 20.2 depending on the estimation method used. When compared to the normative cost of US$ 38.5 per capita, all these estimates of actual PHC expenditures were significantly lower, indicating a shortfall in the funding required to deliver an expanded package of high-quality services to a larger population in line with GoE targets.

**Discussion:**

The study findings underscore the need for increased mobilization of PHC resources and identify opportunities to improve the efficiency of PHC services to meet the GoE’s PHC targets. The data from this study can be a critical input for ongoing PHC financing reforms undertaken by the GoE including transitioning woreda-level planning from input-based to program-based budgeting, revising community-based health insurance (CBHI) packages, reviewing exempted services, and implementing strategic purchasing approaches such as capitation and performance-based financing.

## Introduction

The Government of Ethiopia (GoE) has instituted significant health reforms over the past 15 years to expand primary health care (PHC) services, driven by the Health Sector Transformation Plans (HSTPs) I and II, the Essential Health Services Package (EHSP), the Health Extension Program (HEP), and the expansion of community-based health insurance (CBHI). Updated in 2019, the EHSP outlines nine major components comprising 1,018 evidence-based services to be implemented at each level of care (health posts, health centers, and primary, general, and tertiary hospitals) through different financing mechanisms (free-of-charge or exempted services, cost sharing, or full cost recovery) (1). The country also developed a new HEP roadmap for 2020–2035 to expand access to essential health services at the community level (2). This roadmap sets out plans for establishing community health programs at health centers and primary hospitals, upgrading health posts, improving the professional mix of health post staff, and enhancing the community health information system (2). These GoE policies have been accompanied by significant decentralization. One of the objectives of the HSTP is to improve population health through woreda (district) transformation (3–5). Woreda officials play a central role in realizing national PHC priorities as they are responsible for strategic planning, allocation of resources, and the execution, monitoring, and evaluation of PHC services delivered in facilities and communities (6).

Despite the growing emphasis on strengthening PHC and the advocacy for additional financial investments, ensuring the provision of all services outlined in the EHSP and HEP roadmap will require a significant increase in PHC financing (7). Public funding for the health sector accounted for 8.5% of total general government spending in 2019/20, significantly lower than the 15% target set by the Abuja Declaration (8). In addition, the share of recurrent government spending allocated to facilities delivering PHC services declined drastically from 61% in 2016/17 to 45.1% in 2019/20 (8). Ethiopia also remains heavily reliant on external financing and out-of-pocket health expenditure (OOPHE), with donor sources contributing an estimated 34% and OOPHE constituting 31% of health spending in 2019/20 (8).

Furthermore, while woreda-based planning is intended to establish realistic targets based on available resources, top-down negotiations from the federal and regional levels often result in misalignment and under-performance. Woreda-level budgets typically rely on arbitrary, historical estimates, with many woredas reporting budgets that have been incrementally adjusted from the previous year’s budgets (9). As part of forthcoming provider payment reforms aimed at guiding more output-based and evidence-informed PHC resource allocation, woredas are preparing to transition from input-based budgeting to program-based budgeting.

There are several additional PHC financing reforms underway in Ethiopia to curb cost escalation, enhance equity, and improve quality of care. Health insurance coverage is being expanded with mandatory woreda-level CBHI schemes covering the informal sector since December 2022 and a forthcoming social health insurance (SHI) scheme for the formal sector (10). The Ethiopian Health Insurance Service (EHIS) is revising CBHI benefit packages to align them with the EHSP and standardize them nationwide (11). Additionally, the GoE is reviewing the package of exempted health services and their financing sources (12). The GoE is also piloting a variety of strategic purchasing approaches—capitation is being piloted in Addis Ababa, Amhara, SNNPR, and Oromia, and performance-based financing (PBF) is being tested in Oromia (10).

A pressing issue for Ethiopia as it implements these PHC financing reforms is a clear understanding of PHC service costs (13). There is limited information available on the normative costs of delivering PHC services according to GoE norms and standards (1, 14) or on the actual costs of PHC currently provided in public facilities in Ethiopia (15–17). Cost evidence generated through this study can help inform program-based budgeting and planning at the woreda-level and the prioritization of services included in the CBHI schemes and the exempted health service packages. These cost data can also be used to inform provider payment strategies, ensuring fair rrenumeration that incentivizes the provision of quality services.

The purpose of this study was to calculate and compare the actual cost of delivering PHC services in public sector facilities across seven of Ethiopia’s 11 regional states and its two city administrations with the normative cost of delivering a package of PHC services outlined in the EHSP, and estimate the financial gap. The specific research questions for this study were: (1) what is the actual recurrent cost of delivering PHC services in nine Ethiopian regions, based on a sample of public sector health posts, health centers, and primary hospitals? (2) what is the normative recurrent cost of delivering the package of PHC services specified in the EHSP based on the standard treatment protocols (STPs) and coverage targets defined in the HSTP II? and (3) what is the estimated financial resource gap for delivering PHC services, based on the difference between actual and normative costs?

## Materials and methods

### Costing tool

We used the open access Primary Health Care Costing, Analysis, and Planning (PHC-CAP) Tool to estimate actual and normative costs in nine of Ethiopia’s regions (seven regional states and two city administrations). The PHC-CAP Tool is an activity-based costing tool in Microsoft Excel, which allows users to estimate recurrent actual and normative costs of PHC services provided by health facilities in a geographic area (18). Actual costs are calculated using facility-level input and output data collected from a sample of health facilities and extrapolated to the corresponding universe of facilities in the geographic area of interest (19, 20). Normative costs represent the resources needed to deliver PHC services in the health facilities in the area of interest with reasonable quality and efficiency. Normative costs are derived from costed standard treatment protocols (STPs) developed for the services in a PHC benefits package, considering the estimated populations in need for each service based on population size, estimated disease incidence and prevalence rates, and service coverage targets. The difference between actual and normative costs represents the gap in facility-level recurrent PHC resources. Based on global guidance (21), the PHC-CAP Tool generates reports on the following metrics for both actual and normative costs—subject to data availability: total cost, cost per capita, cost per service/program, cost per input, numbers of inpatient and outpatient services per clinical staff, and average daily service output per clinical staff. In addition to this application in Ethiopia, the PHC-CAP Tool has been used in five other countries including Kenya (22) and Nigeria (23).

### Data collection

We employed purposive sampling to select the PHC facilities for the actual costing. City administrations, woredas, and sub-cities were selected from each of Ethiopia’s three area categories—agrarian, pastoralist, and urban^1^—in consultation with the Federal Ministry of Health (FMOH; Table 1). Within each selected woreda and sub-city, PHC facilities were selected in consultation with regional health bureaus. A total of 20 health posts, 25 health centers, and eight primary hospitals were selected (Table 1). Higher level PHC facilities serve as referral points for lower levels, progressing from health posts to health centers and then to primary hospitals. Health posts concentrate on preventive and promotive services and direct patients to health centers. Health centers focus on outpatient services with limited inpatient care, while primary hospitals provide both outpatient and inpatient care.

**Table 1:** Sample of health facilities by geographic location.

Regional classification	Region/City administration	Health post	Health center	Primary hospital^*^
Agrarian	Amhara	4	4	2
	Oromia	3	4	2
	Sidama	2	2	1
	SNNPR	4	4	2
Pastoral	Afar	2	2	1
	Somali	2	2	
Urban	Addis Ababa		3	
	Dire Dawa	1	2	
	Harari	2	2	
	Total	20	25	8

There were no primary hospitals in selected woredas and sub-cities in Somali, Addis Ababa, Dire Dawa, Harari in 2018/19.

A structured questionnaire was used to capture information from routine health facility records on PHC service outputs, the inputs used to produce the services, and service input prices. Data were collected retrospectively for Ethiopian fiscal year (EFY) 2011, which corresponds to July 8, 2018, to July 7, 2019, in the Gregorian calendar. Two teams were responsible for data collection, each composed of a senior consultant and two data collectors recruited based on previous experience conducting facility surveys. These teams were trained on the data collection instruments. After training, the team piloted the data collection instruments in one health post, one health center, and one primary hospital in the same network in Oromia. The data collection teams conducted interviews with health facility personnel to supplement facility data collection as necessary. Additional data were also obtained from national, regional, city, and woreda health offices including District Health Information System 2 (DHIS2) data, expenditure reports, Program Implementation Plans (PIP), and health service norms and standards. The team collected prices of program drugs and medical supplies from the Ethiopian Pharmaceuticals Supply Service (EPSS). Data for July 8, 2018, to July 7, 2019 were collected from December 2021 to February 2022.

Data were collected on four recurrent input categories: (1) labor (clinical and non-clinical), (2) drugs, (3) medical supplies and laboratory reagents, and (4) utilities and other operational expenditures (e.g., electricity, water). The analysis captured financial costs as well as the costs of in-kind contributions from donors (e.g., for medicines and medical supplies). Above-facility costs (e.g., for supervision and management), capital costs (e.g., equipment, buildings/infrastructure, vehicles), and patient out-of-pocket expenditures outside of the facility (e.g., for transportation and meals) were excluded from this analysis. Data were collected irrespective of funding source, with funding sources including government funds, facility revenue obtained through user fees and CBHI payments to facilities, and resources provided by development partners. Annual and monthly aggregate total labor expenditure for 2018/19 were obtained from administrative records for the sampled facilities. Detailed data on labor costs—including salary (payroll), allowance, and duties—by staff cadre were obtained from facilities for sampled months within the study period (2018/19). Interviews were conducted to collect data on time spent by clinical staff by department for the week preceding the interview, which was used as a parameter to allocate clinical staff time among different departments.

Detailed review of all data collected at each facility was undertaken by the data collection team senior consultants. Data cleaning was done in CS-Pro. Data completeness and outliers were assessed in Stata and questionable values were investigated by the country team.

### Costing approach

The GoE is in the process of developing a definition of PHC specific to Ethiopia. For the purposes of this study, we defined PHC services as the services provided at PHC facilities as per the Lancet Global Health Commission on financing PHC (10).

We calculated total annual costs for each sampled health center and primary hospital by aggregating labor, drug, medical supply and operational costs for five departments:

- Outpatient department (OPD): Curative services that do not require facility admission, including most cases of malaria and tuberculosis (TB).
- HIV/AIDS department (HIV/AIDS): HIV testing services, antiretroviral therapy, and TB services for people living with HIV.
- Maternal and Child Health department (MCH): Non-emergency maternal and child health services such as antenatal care, post-natal care, family planning, and immunization.
- Delivery department (DEL): Basic obstetric care in health posts and health centers and comprehensive obstetric care in primary hospitals.
- Inpatient department (IPD) and Operating room (OR): Services for procedures requiring facility admission including surgeries.

The department heads (primary hospitals) or facility heads (health centers) were asked to allocate clinical staff time use during the week preceding the interview among these departments, including time spent on administration and unspecified activities. Clinical staff salaries, allowances, and duty payments were allocated to each department and to administrative or unspecified activities proportionately to these times. Program drugs and medical supplies clearly used in one of the five departments were allocated accordingly, and those that could not be clearly allocated to one department were allocated in proportion to clinical labor costs. Non-clinical staff time, utilities, and other operational expenditures not directly attributable to one of the five departments were categorized as indirect costs.

The data gathered for health posts were not as extensive as those compiled for health centers and primary hospitals (15). Information on indirect costs in health posts was sourced from the associated health centers and could not be disentangled, although we note that indirect costs in health posts were generally minimal due to the limited presence of non-clinical labor. In addition, staff time allocation data were not collected for health post staff, and available health post costs could not be distributed across departments.

To calculate unit costs per patient, we divided the total cost and department costs by the respective number of patients. Departmental unit costs included indirect expenses like utilities, allocated according to labor costs. They excluded indirect administration costs and the costs of staff (e.g., laboratory, environmental health) not allocated to the five departments. The overall unit costs did include these non-department-specific costs.

To calculate the actual PHC costs in the nine sampled regions, we used three different approaches. In the first two approaches, actual costs per capita were calculated for each health facility sampled by dividing the annual total cost by facility catchment population estimates. In estimate A1, annual total costs were divided by the mid-points of the FMOH reference facility catchment populations and in estimate A2, annual total costs were divided by the catchment population reported by the facilities (Supplementary Table S1). For estimates A1 and A2, simple averages of actual cost per capita were calculated for each facility level and these averages were multiplied by the population of the nine regions to obtain the total actual cost. A limitation of estimates A1 and A2 is that catchment populations are often difficult to establish (19, 20). The differences between estimates A1 and A2 reflect the fact that most facilities reported larger catchment populations than those prescribed by government norms.

In the third approach (estimate B), we estimated the total actual PHC costs in the nine sampled regions by comparing service utilization in sampled facilities with the region-wide utilization data for 2018/19 obtained from the DHIS2. To compare the utilization of different types of services, services were categorized into the five departments (OPD, HIV/AIDS, MCH, DEL, and IPD/OR), and weighted in proportion to the department unit costs in the sample. Expansion factors were calculated separately for each region and facility level by dividing the total weighted utilization in the region or facility by the total weighted utilization in the sampled facilities (in the same region or facility). Total actual PHC costs in the nine sampled regions were estimated by multiplying the actual costs in the sample by the expansion factors. The actual per capita costs were obtained by dividing total actual PHC costs by the total population in the nine sampled regions. A limitation of estimate B is that it relies on utilization data reported in the DHIS2 known to have issues of completeness (24).

For the normative costs, we reviewed the PHC services included in the revised EHSP (Supplementary Table S2). Only PHC services to be delivered in health posts, health centers, and primary hospitals were retained (Supplementary Table S3). Normative costs for these PHC services were estimated based on the STPs for these services, their associated costs, and coverage targets for 2024/25 from the HSTP II for each facility level. For each service, the following information was determined to calculate the normative cost per service at each facility level (health post, health center, and primary hospital):

- Population in need for each service, computed based on the population of each region and city administration and the disease incidence or prevalence rate (i.e., number of expected episodes per target population per year).
- Total drug, diagnostic, and laboratory reagent requirements per service episode and the corresponding unit cost for each.
- Average number of annual encounters per service episode.
- Total human resource requirements, based on the number of minutes required per service by human resource cadre (physician, nurse, or other) and the corresponding salary cost.

The STPs focused on direct costs only. To account for indirect costs that were not included in the STPs but included in the actual costs, such as non-clinical labor, utilities and other operational expenses, an overhead rate was calculated based on our sampled facilities. This overhead was added to the normative human resource, drug, and medical supply costs. The overhead rate for each facility level was determined by dividing the indirect costs by the total costs. This approach assumes linear increases in indirect costs which does not take into account potential economies of scale and scope.

To be consistent with previous normative costing work conducted in Ethiopia, all this information was initially sourced from the FMOH-validated One Health Tool (OHT) dataset used to cost the HSTP II obtained from the FMOH. Following in-depth reviews of population in need estimates for services comprising 95% of total PHC costs, population in need estimates were updated based on population data obtained from the Central Statistics Agency (CSA) of Ethiopia and incidence and prevalence rates obtained from the Institute for Health Metrics and Evaluation Global Burden of Disease (IHME GBD) database (25) and peer-reviewed studies in Ethiopia (Supplementary Table S4). Following this review, population in need estimates were updated with sources for services, comprising 33% of the total PHC package cost (Supplementary Table S4). The team also conducted an additional review of the STPs for high-cost services, comprising 50% of the total PHC package cost. Based on exchanges with the FMOH, the study team made specific changes to STPs which were validated by the FMOH (Supplementary Table S5).

We estimated the financial resource gap for PHC services in the nine sampled regions (Amhara, Oromia, Sidama, SNNPR, Afar, Somali, Addis Ababa, Dire Dawa, and Harari) by subtracting estimates of actual cost per capita from the normative cost per capita. This approach of comparing actual and normative PHC costs per capita to estimate resource requirements has been used in previous work (19, 20). We calculated three distinct PHC resource gaps using the actual per capita estimates A1, A2, and B.

All costs are presented in United States Dollars (US$). The currency exchange rate used for the analysis was 28.44 Ethiopian Birr (ETB) equal to 1 US$ reflecting the rate for 2018/19.

### Sensitivity analysis

Given possible uncertainty in the financial expenditure data collected at the facility level, we conducted one-way sensitivity analyses to assess the influence of varying different parameters on the actual and normative costs per capita. For actual cost per capita, we adjusted clinical labor costs by ±10%, and drug costs, medical supply costs, and indirect costs by ±20% since the data on drugs, medical supplies, and indirect costs are subject to higher levels of uncertainty. For normative costs, we also adjusted indirect costs by ±20% since the indirect costs included in the normative estimates were derived from our actual costs, and we adjusted drug, medical supply, and clinical labor costs by ±10%. For both actual and normative costs, we varied population by 5% given inconsistencies in existing population estimates. In addition to these individual changes, we also calculated best- and worst-case scenarios by simultaneously modifying all these variables.

## Results

### Sample facility characteristics

Staffing patterns in the sampled facilities are displayed in Table 2. On average, the total number of staff at each facility level was two at health posts (almost entirely clinical staff), 65 at health centers (50% clinical, 50% non-clinical), and 169 at primary hospitals (46% clinical staff, 54% non-clinical staff). Staffing at these facilities is primarily determined by government norms. According to these norms, health posts should have two clinical staff (26), health centers should have at least 19 clinical staff (26), and primary hospitals should have a minimum of 59 clinical staff (26). While the clinical staffing in the sampled health posts aligned with the normative requirements, the number of clinical staff in sampled health centers and primary hospitals significantly exceeded minimum requirements.

**Table 2:** Characteristics of sampled facilities by facility level, 2018/19.

	Health post (*N = 20*)	Health center (*N = 25*)	Primary hospital (*N = 8*)
Catchment population			
Mean	6,143	33,646	181,385
Median	6,009	28,914	172,852
Range	2,730–10,556	7,250–81,304	56,583–306,000
Total staff			
Mean	2	65	169
Median	2	54	168
Range	1–5	12–164	117–238
Percent clinical staff			
Mean	NA	50%	46%
Median	NA	47%	47%
Range	NA	34–76%	35–52%
Total number of services			
Mean	7,472	59,526	77,952
Median	6,592	47,852	76,027
Range	317–18,523	10,624–161,962	19,632–175,870
Number of services per clinical staff per day			
Mean	13	9.1	6.2
Median	9.3	7.9	6.6
Range	0.4–39.7	1–37.2	1.2–11.1

Number of services per clinical staff per day is defined as the number of services (outpatient equivalent visits) at a facility in 2018/19 divided by the number of clinical staff at the facility and available working time in days estimated at 226 days; Number of services (outpatient equivalent visits) calculated using the following weights based on the average department unit costs: OPD = 1 outpatient visit, HIV/AIDS = 1.16 outpatient visit, MCH = 0.62 outpatient visit, DEL = 10.2 outpatient visits, and IPD/OR = 9.44 outpatient visits as per Mann et al. (27).

**Figure 1:**
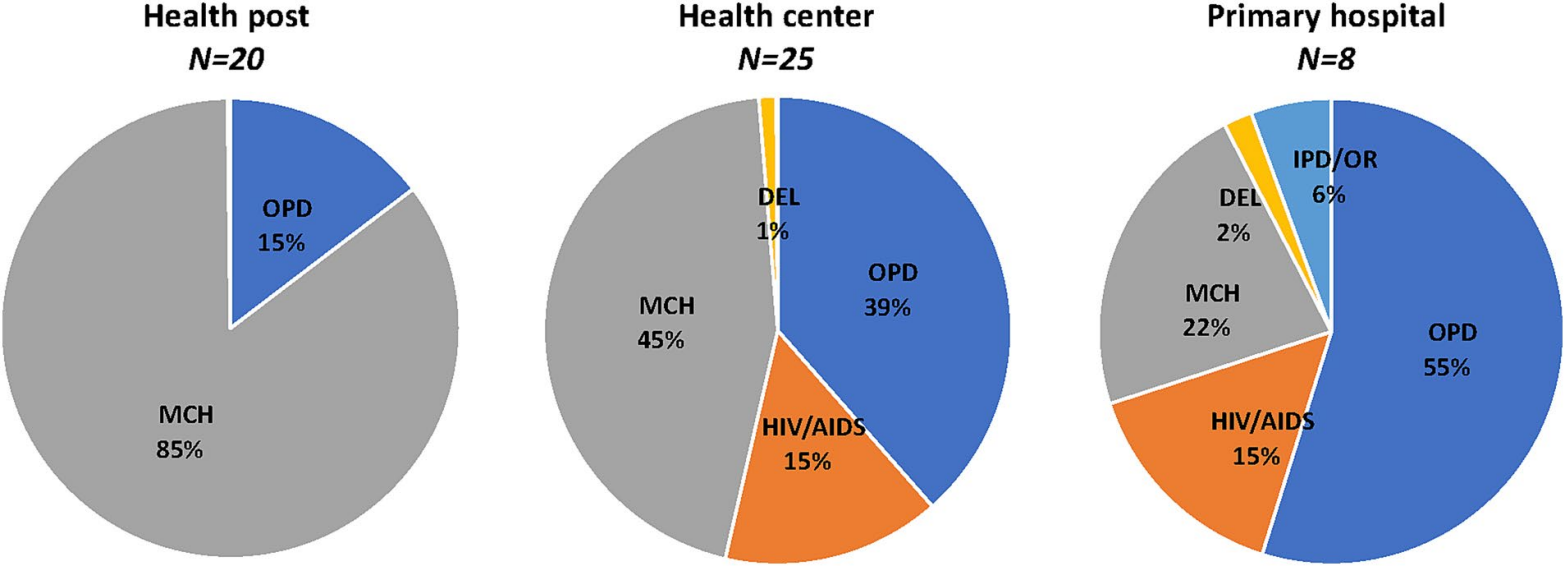
Distribution of services in sampled facilities, 2018/19. Percent distribution of annual mean number of services in sampled facilities. OPD, Outpatient department; MCH, Maternal and child health; DEL, Delivery; and IPD/OR, Inpatient department and operating room. DEL <1% in health posts, HIV/AIDS <1% in health posts, no IPD/OR in health posts, and IPD/OR < 1% in health centers.

Data sourced from the DHIS2 show that on average, 7,472, 59,526, and 77,952, services were provided in health posts, health centers, and primary hospitals, respectively (Table 2). There was considerable variation in the number of services within each facility level, ranging from 317 to 18,523 in health posts, 10,624 to 141,962 in health centers, and 19,632 to 175,870 in primary hospitals. While all hospitals provided services across all five departments (OPD, HIV/AIDS, MCH, DEL, and IPD/OR), not all health centers or health posts did so (Figure 1). Most services provided at all three facility levels were concentrated in the OPD, MCH, and HIV/AIDS departments. Health posts and health centers predominantly delivered services in the MCH department, while in primary hospitals, the largest share of services was in the OPD department.

The daily output of clinical staff also varied by facility level, with higher daily outputs observed for clinical staff in health posts and lower daily outputs for clinical staff in primary hospitals (Table 1). This variation likely stems at least in part from differences in case mixes across healthcare levels, with services provided at primary hospitals requiring more time compared to those provided at health posts. However, there were also notable differences in the number of outpatient equivalent services provided per day by clinical staff across facilities within each facility category, with ranges from 0.4 to 39.7 in health posts, 1 to 37.2 in health centers, and 1.2 to 11.1 in primary hospitals.

### Actual costs in sample facilities

**Figure 2:**
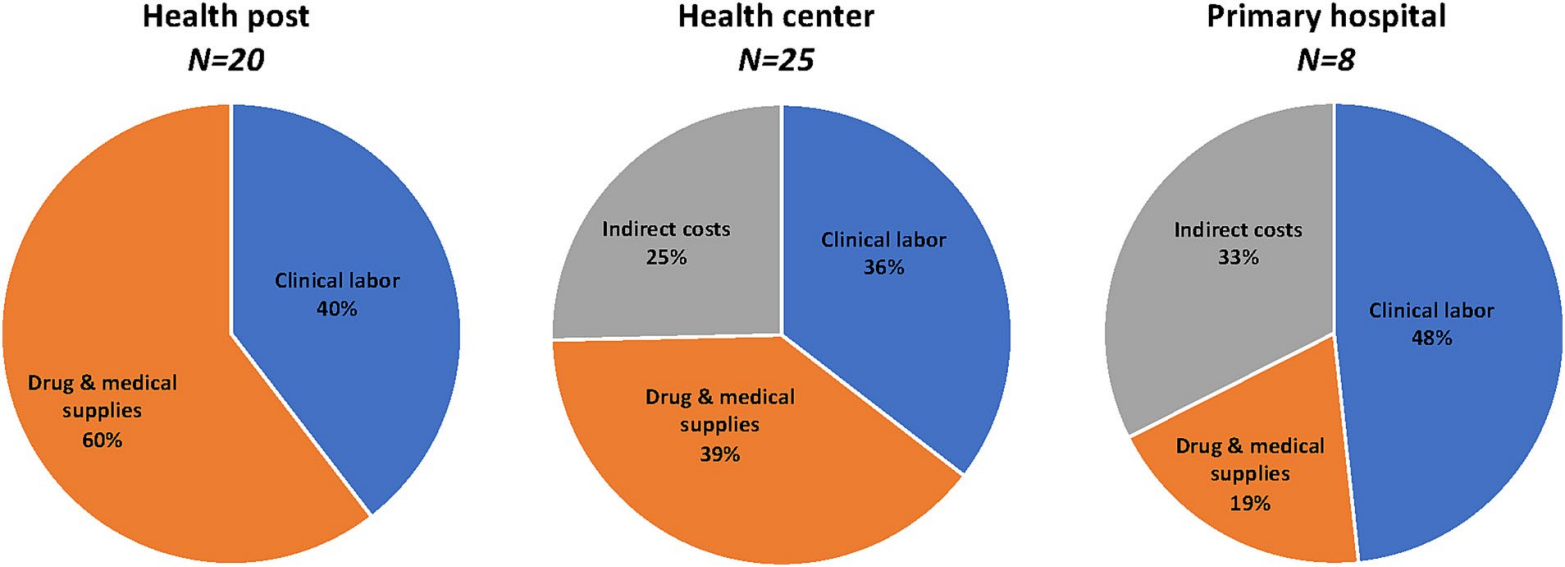
Distribution of costs in sampled facilities, 2018/19. Percent distribution of annual mean costs in sampled facilities. *Clinical labor*, includes labor expended on clinical services by doctor, nurse, midwife, other health worker, social worker, technician. *Indirect costs*, includes costs of labor expended on administrative tasks, as well as utilities and other recurrent costs. Indirect costs for health posts included in health centers to which they are attached.

Total PHC service delivery costs for the sampled health facilities are displayed in Table 3. On average, the total annual cost of delivering PHC services was US$ 11,532 in health posts, US$ 254,340 in health centers, and US$ 634,354 in primary hospitals. Clinical labor costs accounted for 40% of costs in health posts, 36% in health centers, and 48% in primary hospitals (Figure 2). Drugs and medical supplies represented 60% of costs in health posts, 39% in health centers, and 19% in primary hospitals. Indirect costs constituted 25% of costs in health centers and 33% in primary hospitals. Indirect costs in health posts are included in the costs of the health centers to which they are attached.

**Table 3:** Actual costs in sampled facilities by facility level, US$, 2018/19.

	Health post (*N = 20*)	Health center (*N = 25*)	Primary hospital (*N = 8*)

Total costs			
Mean	11,532	254,340	634,354
Median	6,566	183,271	594,330
Range	934–40,746	68,860–832,647	505,208–970,720
Total clinical labor costs			
Mean	4,563	90,150	306,400
Median	2,711	61,092	313,624
Range	632–37,376	22,077–297,875	261,959–356,963
Total drug & medical supply costs			
Mean	6,970	99,711	122,110
Median	2,092	68,316	83,957
Range	0–40,114	1,752–326,781	14,764–400,855
Total indirect costs			
Mean		64,480	205,844
Median		40,159	198,186
Range		12,333–207,990	104,719–361,654

Clinical labor includes labor expended on clinical services by doctor, nurse, midwife, other health worker, social worker, technician. Indirect costs includes costs of labor expended on administrative tasks, as well as utilities and other recurrent costs. Indirect costs for health posts are included in health centers to which they are attached and could not be separated.

Table 4 presents total PHC costs by department in sampled health centers and primary hospitals (actual costs by department were not estimated for health posts since staff time allocation was not collected from health posts). On average, the costs incurred across the five departments made up 75% of total costs in health centers and 68% in primary hospitals. The distribution across departments varied by facility level with the highest costs incurred by OPD, HIV/AIDS, and MCH in health centers, and OPD and IPD/OR in primary hospitals. On average administrative and other indirect costs were US$ 40,244 and US$ 24,236 in health centers and US$ 121,742 and US$ 84,103 in primary hospitals. The proportions of administrative and other indirect costs were slightly lower in health centers (16 and 10%) than in primary hospitals (19 and 13%).

**Table 4:** Actual costs in sampled health centers and primary hospitals by facility level and department, US$, 2018/19.

	Health center (*N = 25*)	Primary hospital (*N = 8*)

Total costs		
Mean	254,340	634,354
Median	183,271	594,330
Range	68,860–832,647	505,208–970,720
OPD costs		
Mean	55,862	137,625
Median	48,699	137,694
Range	14,846–150,064	74,231–186,457
HIV/AIDS costs		
Mean	54,638	46,672
Median	31,067	22,923
Range	0–234,898	0–225,070
MCH costs		
Mean	39,615	46,056
Median	31,928	37,668
Range	9,131–107,888	14,037–93,309
DEL costs		
Mean	14,545	31,581
Median	6,768	36,734
Range	2,639–64,386	13,055–48,154
IPD/OR costs		
Mean	7,264	118,411
Median	0	101,405
Range	0–62,898	55,576–209,135
Other service costs		
Mean	17,936	48,164
Median	10,466	46,645
Range	1,558–63,780	30,454–66,871
Administration costs		
Mean	40,244	121,742
Median	29,865	126,652
Range	6,543–112,681	58,672–155,002
Other indirect costs		
Mean	24,236	84,103
Median	13,579	58,879
Range	1,558–63,780	30,454–66,871

OPD, Outpatient department; MCH, Maternal and child health; DEL, Delivery; and IPD/OR, Inpatient department and operating room. Other service costs includes staff costs for staff (e.g., laboratory, environmental health), which were not allocated to the five departments. Administration costs includes cost of labor expended on administrative tasks and administrative supplies. Other indirect costs includes utilities and other recurrent costs. Actual costs by department were only estimated for health centers and primary hospitals since information on staff time allocation was not collected for health post staff.

Unit costs per patient by department for the sampled facilities are shown in Table 5. Health posts had an average unit cost of US$ 2.9, health centers US$ 5, and primary hospitals US$ 11.6. In health centers, the range was US$ 2.4 in MCH to US$ 442.7 in IPD/OR. In primary hospitals, departmental average costs ranged from US$ 4.5 in MCH to US$ 52.2 in IPD/OR. Median unit costs, both overall and by department, were typically lower than mean costs at all care levels. This discrepancy was especially pronounced in the DEL and IPD/OR departments of health centers and the MCH department of primary hospitals, where a few high values skewed the data. The reason for these outliers remains unclear; they may reflect reporting errors, underutilization, or inefficiencies.

**Table 5:** Per patient costs in sampled facilities by facility level and department, US$, 2018/19.

	Health post	Health center	Primary hospital	All levels
	*(N = 20)*	*(N = 25)*	*(N = 8)*	*(N = 53)*
OPD unit cost				
Mean		4.5	5.5	4.7
Median		2.7	3.8	3.3
Range		0.7–18.4	1.3–17.6	0.7–18.4
HIV/AIDS unit cost				
Mean		5.6	5.0	5.5
Median		5.2	3.4	4.9
Range		0.1–12.9	1.3–13.6	0.1–13.6
MCH unit cost				
Mean		2.4	4.5	2.9
Median		1.9	2.3	2.0
Range		0.2–11.7	1.3–18.6	0.2–18.6
DEL unit cost				
Mean		53.8	32.3	48.6
Median		22.3	25.4	24.6
Range		7.6–241.5	10.9–77.5	7.6–241.5
IPD/OR unit cost				
Mean		442.7	52.2	234.4
Median		219.1	35.0	42.3
Range		0.3–1132.1	18.8–125.7	0.3–1132.1
Overall unit cost				
Mean	2.9	5.0	11.6	5.2
Median	1.2	3.9	9.7	3.9
Range	0.1–15.4	1.1–14.78	3.2–32.3	0.1–32.3

Unit costs per patient in each facility were calculated by dividing total and departmental costs by the corresponding number of patients. Departmental unit costs account for indirect expenses such as utilities, apportioned based on labor costs. These costs do not include indirect administrative costs and costs of staff (e.g., laboratory, environmental health) not allocated to the departments. Overall unit costs include non-department-specific costs.

### Sources of financing in sample facilities

**Figure 3:**
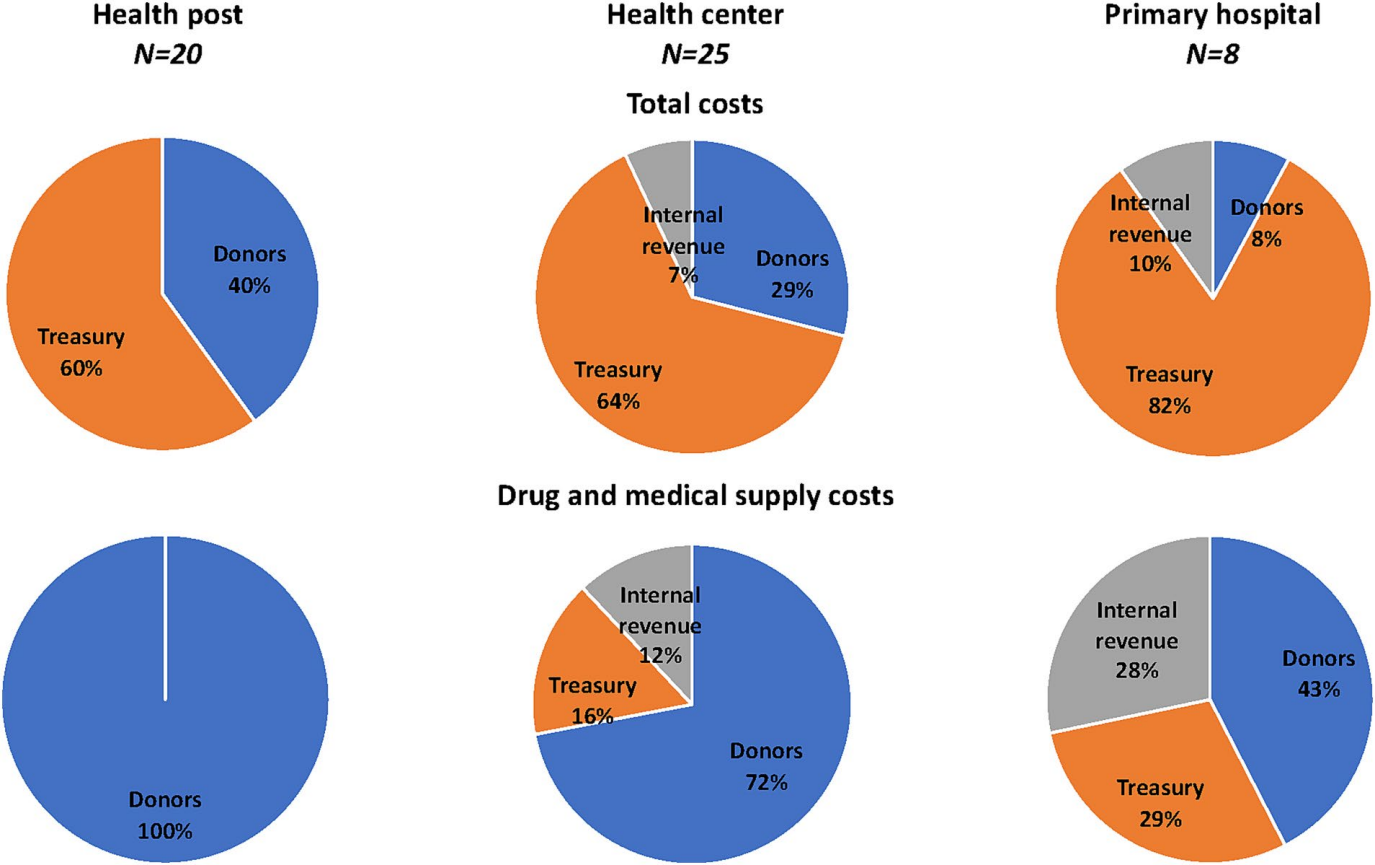
Sources of financing in sampled facilities, 2018/19. Percent distribution of financing sources in sampled facilities for total costs and drug and medical supply costs. Funding sources include government funds (*Treasury*), facility revenue obtained through user fees and community-based health insurance (CBHI) facility payments (*Internal revenue*), and resources provided by development partners (*Donors*).

Figure 3 displays PHC service delivery total annual costs and drug and medical supply costs by financing source. The Treasury which covers labor costs at all facility levels, was the largest financing source at all facility levels. Drugs and medical supplies were primarily financed by donors, with some contributions from the Treasury and internal revenue. Donor-funded programmatic drugs, including those for HIV/AIDS, family planning, TB, and malaria, comprised 100% of drug costs in health posts, 72% in health centers, and 43% in primary hospitals. Health facilities’ internal revenues, collected through user fees and CBHI payments, accounted for almost 12% of drug costs in health centers and nearly 28% in primary hospitals.

### Actual costs and normative costs in network of facilities

Total annual actual costs extrapolated from sampled facilities to all health posts, health centers, and primary hospitals in the nine sampled regions (Amhara, Oromia, Sidama, SNNPR, Afar, Somali, Addis Ababa, Dire Dawa, and Harari) are shown in Table 5. Total actual costs were estimated to range between US$ 142.3 and US$ 266.4 million in health posts, between US$ 237.3 and US$ 866.6 million in health centers, and between US$ 57.2 and US$ 732.7 million in primary hospitals depending on the extrapolation method used. Comparing actual cost estimates with the normative cost estimates, which incorporate a more comprehensive package of services as specified in the EHSP and expanded service utilization as per HSTP II coverage targets for 2024/25 (EFY 2017), shows that PHC services are considerably underfunded. Overall gaps were significant for all facility levels regardless of the actual cost extrapolation method used though the estimation methods based on catchment populations (A1 and A2) suggest there may be excess clinical labor at all facility levels (estimate A1) or at lower-level facilities (estimate A2).

Actual costs per capita ranged from US$ 4.7 to US$ 20.2 depending on the extrapolation method used, compared to the normative cost of US$ 38.5 per capita required to meet PHC service utilization targets outlined in the HSTP II (Table 6). This amounts to an annual per capita resource gap ranging between 48 and 88%. The actual cost per capita was lower than the normative cost per capita at all levels of care, resulting in a resource gap between 53 and 75% at health posts, between 39 and 83% at health centers, and between 27 and 96% at primary hospitals.

**Table 6:** Actual costs and normative costs in network of facilities by facility level, 2018/19.

	Health post	Health center	Primary hospital	Total
Population (million)				92.4
Percent of actual services in network captured in sample	0.1	2.3	10.4	
Actual costs (US$ million)				
*Estimate A1*				
Total	266.4	866.6	732.7	1865.6
Clinical labor	105.4	307.7	353.9	766.9
Drugs and medical supplies	161.0	347.7	141.0	649.8
Indirect	0.0	211.2	237.7	448.9
*Estimate A2*				
Total	217.7	777.5	390.8	1386.0
Clinical labor	86.9	276.5	191.0	554.4
Drugs and medical supplies	130.8	304.8	75.3	510.9
Indirect	0.0	196.2	124.5	320.7
*Estimate B*				
Total	142.3	237.3	57.2	436.8
Clinical labor	69.0	73.2	27.7	169.9
Drugs and medical supplies	73.4	112.1	12.4	197.9
Indirect	0.0	52.0	17.0	69.0
Normative costs (US$ million)				
Total	571.6	1416.4	1567.0	3554.9
Clinical labor	79.9	177.1	277.7	534.7
Drugs and medical supplies	491.7	894.1	780.8	2166.6
Indirect	0.0	345.2	508.5	853.6

Network consists of all health posts, health centers, and primary hospitals in nine sampled regions (Amhara, Oromia, Sidama, SNNPR, Afar, Somali, Addis Ababa, Dire Dawa, and Harari). Actual cost estimates calculated as follows: estimate A1, total annual actual costs in sampled facilities divided by FMOH reference catchment populations and simple averages of actual cost per capita calculated for each facility level and multiplied by populations of the nine regions; estimate A2, total annual actual costs in sampled facilities divided by catchment populations reported by facilities and simple averages of actual cost per capita calculated for each facility level and multiplied by populations of the nine regions; estimate B, total actual costs in sample expanded to nine sampled regions in proportion to utilization. Normative cost estimate for PHC services specified in the Essential Health Services Package (EHSP) with coverage targets defined in the Health Sector Transformation Plan (HSTP) II. Clinical labor includes labor expended on clinical services by Doctor, Nurse, Midwife, Other health worker, Social worker, and Technician. Indirect costs includes costs of labor expended on administrative tasks, as well as utilities and other recurrent costs. Indirect costs for health posts are included in health centers to which they are attached.

Table 7 also shows actual and normative costs per capita and the gap range by facility level and department where data were available (i.e., it was not possible to estimate actual cost per capita by department for health posts). When comparing actual and normative costs by department, one difference between the actual and normative per capita costs by department is that other services, such as costs for laboratory staff and environmental health staff were not allocated to different departments in the actual costs, while these were included in the normative department costs. However, distributing these actual support service costs across departments would not change the conclusion that there is a large financing gap in almost every department in health centers and hospitals. The exception is HIV/AIDS in health centers, which appears to be excessively financed in two of the actual cost estimates. While MCH, OPD, and HIV/AIDS were the departments with the largest financing gaps in hospitals, OPD and IPD/OR were the departments with the largest financing gaps in health centers. Actual costs by department were not estimated for health posts since staff time allocation was not collected from these facilities. Normative costs for health posts were concentrated in OPD and MCH, which corresponds well to current utilization, although health posts appear to have under-reported HIV/AIDS services in the DHIS2.

**Table 7:** Annual actual and normative PHC costs per capita and corresponding gaps in network of facilities, by facility level and department, US$, 2018/19.

Facility level and department	Actual cost per capita			Normative cost per capita	Gap
	*Estimate A1*	*Estimate A2*	*Estimate B*		
*Health post*					
OPD				3.3	
HIV/AIDS				0.6	
MCH				2.3	
DEL				0.0	
IPD/OR				0.1	
Total	2.9	2.4	1.5	6.2	53–75%
*Health center*					
OPD	2.2	1.8	0.6	5.8	62–90%
HIV/AIDS	2.0	1.6	0.6	1.3	0–52%
MCH	1.6	1.6	0.5	2.3	32–79%
DEL	0.5	0.5	0.1	0.8	36–84%
IPD/OR	0.2	0.2	0.0	1.4	84–97%
Other service	0.6	0.6	0.2		
Indirect	2.3	2.1	0.6	3.7	39–85%
Total	9.4	8.4	2.6	15.3	39–83%
*Primary hospital*					
OPD	1.7	0.9	0.1	5.5	69–97%
HIV/AIDS	0.6	0.3	0.1	2.0	71–97%
MCH	0.6	0.3	0.0	1.2	53–96%
DEL	0.4	0.2	0.0	0.6	37–95%
IPD/OR	1.5	0.8	0.1	2.0	27–95%
Other service	0.6	0.3	0.0		
Indirect	2.6	1.3	0.2	5.5	53–97%
Total	7.9	4.2	0.6	17.0	53–96%
Total	20.2	15.0	4.7	38.5	48–88%

Network consists of all health posts, health centers, and primary hospitals in nine sampled regions (Amhara, Oromia, Sidama, SNNPR, Afar, Somali, Addis Ababa, Dire Dawa, and Harari). Actual cost estimates calculated as follows: estimate A1, total annual actual costs in sampled facilities divided by FMOH reference catchment populations; estimate A2, total annual actual costs in sampled facilities divided by catchment populations reported by facilities; estimate B, total actual costs in sample expanded to nine sampled regions in proportion to utilization, then divided by total population in the nine regions. Normative cost estimate for PHC services specified in the Essential Health Services Package (EHSP) with coverage targets defined in the Health Sector Transformation Plan (HSTP) II. Indirect costs for health posts are included in health centers to which they are attached. OPD, Outpatient department; MCH, Maternal and child health; DEL, Delivery; and IPD/OR, Inpatient department and operating room. Other service includes staff costs for staff (e.g., laboratory, environmental health) which were not allocated to the five departments in the actual costs but are included in normative department costs. Indirect includes costs of labor expended on administrative tasks, as well as utilities and other recurrent costs. Actual costs by department were only estimated for health centers and primary hospitals since information on staff time allocation was not collected for health post staff. Gaps are expressed as a percentage of need by comparing the normative costs per capita with the various estimates of actual costs per capita.

### Sensitivity analysis

In the best- and worst-case scenarios obtained by simultaneously varying clinical labor costs, drug costs, medical supply costs, indirect costs, and population size, the actual cost per capita estimates ranged from US$ 16.2 to US$ 24.6 (estimate A1), US$ 12 to US$ 18.3 (estimate A2), and US$ 3.8 to US$ 5.8 (estimate B; Supplementary Figure S1). The normative cost per capita ranged from US$ 32.1 to US$ 45.5 (Supplementary Figure S2; Table 7).

## Discussion

The findings from this study show that the average actual PHC cost per capita in nine Ethiopian regions in 2018/19 was US$ 4.7 to US$ 20.2 overall, with US$ 1.5 to US$ 2.9 in health posts, US$ 2.6 to US$ 9.4 in health centers, and US$ 0.6 to US$ 7.9 in primary hospitals. When compared to the normative cost estimate of US$ 38.5 per capita, the actual PHC expenditures are significantly lower than what would be needed to deliver high-quality services. Based on the HSTP II targets for 2024/25 at each facility level, the overall resource gap ranged from 48 to 87%, with gaps of 53 to 75% in health posts, 39 to 83% in health in centers, and 53 to 96% in primary hospitals.

Recent studies in Afghanistan (20), Kenya (22), and Nigeria (23) have also explored the actual and normative costs of delivering PHC service packages. In line with the findings from this study in Ethiopia, these studies also highlight significant gaps between current PHC resources and the funds needed to deliver quality care to all in need as well as the opportunities to reduce this funding gap through efficiency gains.

Our study’s actual cost estimates are consistent with previous empirical costing work conducted in health facilities in Ethiopia. A study examining the actual cost of PHC services in Amhara, Oromia, Benshangul-Gumuz, Somali, Addis Ababa, and Dire Dawa in 2013/14 (EFY 2006) found that the average per capita expenditure was US$ 9.1 for health centers and 4.9 for primary hospitals and US$ 6.5 for health centers and US$ 4.9 for primary hospitals when accounting for catchment population outliers (16).

Our study’s normative cost estimates of US$ 29.2 per capita are lower than EHSP resource requirement estimates of Hailu et al. (14), which ranged from US$ 54.3 to US$ 107.4 per capita in 2025 depending on the service coverage levels assumed. There are several reasons for this difference. While this study focused only on PHC services within the EHSP, Hailu and colleagues’ study included all 1,018 services. Their analysis also considered costs not captured in this study, such as capital costs for health facilities and equipment, as well as facility-level and above-facility costs for logistics, health information systems, health financing, governance, and program management.

When compared to the multi-country analyses of PHC per capita costs that feature Ethiopia, this study’s actual per capita cost estimates tend to be lower. The Institute for Health Metrics and Evaluation (IHME) Global Burden of Disease (GBD) estimated Ethiopia’s PHC expenditure per capita to be US$ 17.20 in 2017 (28), while the World Health Organization (WHO) reported it to be US$ 21.12 in 2019 (29). There are considerable methodological differences between these studies and our work and both IHME GBD and WHO estimates rely on country-reported health expenditure data that includes private providers and above service level expenditures, which this study does not consider.

Our study’s normative cost per capita estimates are lower than those reported by WHO but consistent with those of the DCP3. WHO estimated an average per capita cost of US$ 65 for low-income countries by 2030 (30), and the DCP3 reported a 2015 per capita cost of US$ 42 for an essential package and US$ 76 for a more comprehensive package (31). Both WHO and DCP3 analyses factored in significant health system investments not included in our study, and there are differences across the studies in how PHC is defined and the numbers of PHC interventions that are costed.

Besides shedding light on Ethiopia’s financial gap for PHC, this study also offers data on the cost per PHC service at various facilities. Health posts had an average unit cost of US$ 2.9, health centers US$ 5, and primary hospitals US$ 11.6. These average costs per patient are consistent with average cost per PHC services at PHC facilities for Ethiopia (15, 32) and other countries in sub-Saharan Africa (33) which typically range between US$ 5 and US$ 10. However, variations in cost within the same level of care suggest potential inefficiencies at some facilities.

Our findings also indicate that while labor costs in health posts, health centers, and primary hospitals were financed through government revenues, drugs and medical supplies relied primarily on donor funding. This reliance on donor-funded programmatic drugs, including those for HIV/AIDS, family planning, tuberculosis, and malaria, poses a significant challenge to the sustainability of PHC services (7, 34). Donor-funded drugs accounted for all drug costs in health posts, over three-quarters of the costs in health centers, and nearly half of the costs in primary hospitals. Facility internal revenue collected through user fees and CBHI payments funded almost a third of drug costs in primary hospitals but unfortunately, we were unable disaggregate facility revenues to assess the extent of OOPHE by patients in the facilities sampled. Facility internal revenue collected through user fees and CBHI payments funded almost a third of drug costs in primary hospitals, however, we were unable disaggregate facility revenues to assess the extent of OOPHE by patients in the facilities sampled.

### Policy implications

The FMOH projects health resources from all sources to range from US$ 41.2 to US$ 53.3 per capita in 2024/25 (4). Our normative PHC cost estimate of US$ 38.5 per capita represents 93% of the low and 72% of the high resource projections. This leaves US$ 2.7 and US$ 14.8 per capita for non-PHC health costs under the low and high projections, respectively. To close the PHC resource gap, the government will need to increase health expenditure.

The recent Lancet Global Health Commission recommended more strategic purchasing of PHC services, including an explicitly defined and appropriate benefits package, a shift from input-based budgeting to output-based budgeting, and a blended provider payment mechanism that directs money to PHC, such as capitation (13). Currently, Ethiopia is piloting the capitation provider payment model in SNNPR, Oromia, Amhara, and Addis Ababa regions (10). This study’s cost data can be used to inform capitation provider payment formulae and services to be purchased through capitation. This study’s cost data can more broadly be used to inform EHSP prioritization and CBHI restructuring, including inclusion of more cost-effective PHC services, and decision-making on rationalizing exempted health services across regions. Our data can also be used to inform the development of woreda budgets as woredas shift from historical line-item budgeting to program-based budgeting. In the future, woredas can consider routinizing the collection and analysis of input, output, cost, and epidemiological data and using the PHC-Costing and Analysis Platform (PHC-CAP) Tool to monitor facility performance and improve midterm planning, annual planning, and budget preparation to ensure adequate PHC budget allocations based on resource needs.

While substantial financial investments are crucial for expanding quality PHC service provision in Ethiopia, financial resources alone will not address some of the fundamental causes of PHC underperformance (35). This study does not assess some of the key system-level characteristics (e.g., governance and leadership and goals of the PHC system), nor does it assess some of the major PHC inputs required (e.g., the availability of drugs and supplies), or service delivery processes (e.g., provider competence and availability and quality of care). Several studies have raised concerns about the levels of quality of care of services provided in Ethiopia for example (36–40). Our study does offer insights into some PHC inputs, suggesting that additional resource requirements could be partly offset by improving the efficiency of existing resources.

One area for improvement is the productivity of clinical staff in service provision. Significant variations in daily output per staff were noted within each facility level with some facilities having very low numbers of services per clinical staff per day, indicating inefficiencies. The performance-based financing pilot currently being conducted in Oromia might consider these findings to improve daily output per clinical staff as one of the results for payment (10). For primary hospitals in particular, the average daily output per clinical staff of 6.2 is somewhat higher than previous studies (27) but remains low. This low productivity may result from an imbalance between service demand and high staff numbers in primary hospitals. Both health centers and primary hospitals had significantly higher staffing levels than prescribed in government guidelines. These findings are consistent with previous work showing the significant variability in health worker productivity in Ethiopia (41, 42). Limiting the number of clinical staff to the standards set might also help to improve their productivity.

The findings also suggest significant underutilization of services in certain facilities, as evidenced by the considerable variation in the number of services provided across facilities at each level of care. There was almost a 10-fold difference in the number of services provided between primary hospitals with the lowest and highest service provision. This difference was more than 10-fold among health centers and almost 60-fold among health posts. Extreme values observed for certain unit costs, such as the unit cost for IPD/OR in health centers in Addis Ababa, also indicate potential underutilization. While there could be other explanations, such as reporting errors, service underutilization cannot be ruled out. Previous studies have identified underutilization of PHC facilities as an issue in Ethiopia (27), but this study does not provide information on the reasons for these differences. Self-referral of patients to more distant, higher-level facilities instead of using closer lower-level facilities has been identified as an issue in Ethiopia (43). To address bypassing behaviors, to access free healthcare via CBHI, members must adhere to the referral process (44)—those who skip health centers and go directly to hospitals without a referral letter will not receive reimbursement from the scheme. As CBHI coverage expands and with the roll out of the SHI if it has similar restrictions, any existing bypassing is likely to decrease in frequency.

### Limitations

These study results provide valuable insights into the actual costs of PHC services in nine Ethiopian regions, as well as the costs of providing the PHC services delineated in the EHSP to all those who need them. However, the study has several limitations that should be considered when interpreting the results. Firstly, geographic areas and health facilities within the regions included in this analysis were purposively selected in consultation with the FMOH and regional health bureaus. Given security concerns, the accessibility of facilities for data collectors was a consideration and resulted in some clustering of the geographic areas selected. Secondly, the estimation of actual costs relied on output, input, and price data of variable quality. We used service utilization data reported in the DHIS2 to estimate costs and to extrapolate cost estimates to the sampled regions and these data have quality and completeness issues. We also used estimates of catchment populations to extrapolate actual cost estimates to the sampled regions and these are difficult to estimate (19, 20). Our use of a combination of actual data extrapolation methods aims to offer a range of insights despite these data limitations. Thirdly, the study focuses on public facilities and recurrent facility-level PHC costs, excluding capital costs and above-facility costs such as supply chain costs for example. Fourthly, the normative cost estimates were constructed under the assumption that disease burden is uniform across Ethiopia which is unlikely to be the case. However, regional disease incidence and prevalence rates for many conditions are not available.

### Future research

The data obtained from this study can be used to conduct further analyses to inform PHC prioritization and resource allocation decisions. These “what if” analyses based on our results can be used to estimate a range of scenarios for implementing various PHC service delivery packages at different coverage levels, taking into account services provided at different health facility levels, and comparing associated costs and financial implications.

To enhance understanding of PHC costs and service provision in Ethiopia, additional data collection and analysis would be helpful. It would be beneficial to collect data from private providers, health facilities operated by faith-based organizations, and non-governmental organizations to capture the entirety of PHC service providers. Moreover, detailed information on human resources is crucial, including data on absenteeism, idle staff time, and service quality. Efficient resource use and cost drivers could be better assessed by collecting additional data on staff numbers relative to health service demand and identifying inefficiencies in resource use.

During this study, the data collection process highlighted several issues that require attention for improved future actual and normative PHC cost estimation. Future cost analyses would benefit from the availability of reliable electronic expenditure data and comprehensive and up-to-date service volume statistics. This would reduce the time needed for primary data collection at health facilities, where data may be incomplete or unavailable, such as for program drugs consumption (15) and allow for regular analyses to guide resource allocation.

## Conclusion

This study provides valuable information on PHC costs and resource requirements in Ethiopia and the gap between these two. The study calls for increased PHC resource mobilization to meet the GoE’s current PHC targets as defined in the EHSP and the HSTP II and identifies opportunities to improve the overall efficiency of PHC services. The data from this study can be a critical input to the PHC financing reforms currently being undertaken by the government to increase strategic health purchasing, improve woreda-level planning and budgeting, and ensure more sustainable financing for PHC.

## Data availability statement

The raw data supporting the conclusions of this article will be made available by the authors, without undue reservation.

## Author contributions

CG and AA designed the study. AA, EA, and WM led data collection, validation, and interpretation. RM, MO, and LT conducted data analysis on costs and service utilization. SA, ED, TM, and DW provided critical revisions and inputs on the relevance of the findings to policy reforms. MO, LT, RM, and CG wrote the first draft of the manuscript. All authors contributed to the article and approved the submitted version. Aside from AA and CG, authors are listed in alphabetical order.

## Footnotes

^1^Per government guidelines, health facilities serve varying population sizes: health posts handle 3,000–5,000 people in pastoralist and agrarian settings respectively; health centers serve 25,000–40,000 in rural and urban areas; and primary hospitals serve 60,000–100,000 individuals (2). In pastoralist settings, health posts also provide mobile services where health extension workers provide services in communities.
