# Supplementary material for: Costs and resource needs for primary health care in Ethiopia: evidence to inform planning and budgeting for universal health coverage
Source: Front Public Health. 2023 Dec 19;11:1242314. doi: 10.3389/fpubh.2023.1242314 (PMC10762776; doi:10.3389/fpubh.2023.1242314)
Supplement: Supplementary file 1 [file Table_1.docx]

Supplementary Material

Costs and resource needs for primary health care in Ethiopia: evidence to inform planning and budgeting for universal health coverage

Abebe Alebachew, Engida Abdella, Samuel Abera, Ermias Dessie, Tesfaye Mesele, Workie Mitiku, Rodrigo Muñoz, Marjorie Opuni, Lyubov Teplitskaya, Damian G. Walker, Colin Gilmartin^5*^

*** Corresponding author:** Colin Gilmartin: cgilmartin@msh.org

# Supplementary Figures and Tables

## Supplementary Figures

**Figure S1. Sensitivity analysis for actual PHC costs per capita in network of facilities**

**Figure S1.** Network consists of all health posts, health centers, and primary hospitals in nine sampled regions (Amhara, Oromia, Sidama, SNNPR, Afar, Somali, Addis Ababa, Dire Dawa, Harari). A*ctual cost* estimates calculated as follows: estimate A1, total annual actual costs in sampled facilities divided by Federal Ministry of Health reference catchment populations; estimate A2, total annual actual costs in sampled facilities divided by catchment populations reported by facilities; estimate B, total actual costs in sample expanded to nine sampled regions in proportion to utilization, then divided by total population in the nine regions.

**Figure S2. Sensitivity analysis for normative PHC costs per capita in network of facilities**

**Figure S2.** Network consists of all health posts, health centers, and primary hospitals in nine sampled regions (Amhara, Oromia, Sidama, SNNPR, Afar, Somali, Addis Ababa, Dire Dawa, Harari). *Normative cost* estimate for PHC services specified in the Essential Health Services Package (EHSP) with coverage targets defined in the Health Sector Transformation Plan (HSTP) II.

## Supplementary Tables

**Table S1. Facility catchment populations by facility level**

| **Facility level** | **FMOH reference catchment population ranges** | **Facility reported catchment population ranges** |
| --- | --- | --- |
| Health post | 3,000–5,000 | 2,730–10,556 |
| Health center (rural) | 15,000–25,000 |  |
| Health center (urban) | 40,000 |  |
| Health center (all) |  | 7,250–81,304 |
| Primary hospital | 60,000–100,000 | 56,583–306,000 |

**Table S1.** *FMOH*, Federal Ministry of Health

**Table S2. Major components of revised Essential Health Services Package (EHSP)**

|  | **Revised EHSP components** |
| --- | --- |
| 1. | Reproductive, maternal, child, and adolescent health |
| 2. | Major communicable diseases |
| 3. | Non-communicable diseases |
| 4. | Surgical and injury care |
| 5. | Emergency and critical care |
| 6. | Neglected tropical diseases |
| 7. | Hygiene and environmental health services |
| 8. | Health education and behavior change communication services |
| 9. | Multi-sectoral nutrition interventions |

**Table S2.** Source: Federal Ministry of Health

**Table S3. List of normative PHC services and costs per case, USD**

| **Normative service** | **Community** | **Outreach** | **Health Center** | **Primary Hospital** |
| --- | --- | --- | --- | --- |
| 1001. Syphilis screening (pregnant women) |  |  | 0.74 | 0.74 |
| 1004. Tetanus toxoid (pregnant women) |  | 0.45 | 0.29 | 0.29 |
| 1005. Antenatal corticosteroids for preterm labor |  |  | 15.51 | 15.51 |
| 1006. Antibiotics for preterm or prolonged PROM |  |  | 14.39 | 14.39 |
| 1009. Nutritional care and support for pregnant and lactating women in emergencies |  |  | 3.78 |  |
| 1011. Clean birth environment | 1.88 |  |  |  |
| 1013. Newborn sepsis - Full supportive care |  |  |  | 126.10 |
| 1014. PMTCT |  |  | 27.56 | 27.56 |
| 1015. Neonatal resuscitation |  |  | 10.76 | 10.76 |
| 1016. Kangaroo mother care |  |  | 39.80 | 39.80 |
| 1017. Breastfeeding counselling and support | 0.64 |  | 1.11 |  |
| 1018. Complementary feeding counselling and support | 0.28 |  | 0.48 |  |
| 1021. Measles vaccine | 0.47 | 0.44 | 0.51 | 0.51 |
| 1022. Insecticide treated materials | 2.96 | 2.96 |  |  |
| 1025. Hib vaccine | 10.19 | 10.19 | 10.29 | 10.29 |
| 1026. Pneumococcal vaccine | 9.31 | 9.31 | 9.51 | 9.51 |
| 1027. ORS |  |  | 4.12 |  |
| 1028. Antibiotics for treatment of dysentery |  |  | 1.41 | 19.39 |
| 1029. Zinc (diarrhea treatment) |  |  | 0.69 |  |
| 1030. Pneumonia treatment (children) |  |  | 8.68 |  |
| 1031. Vitamin A for measles treatment (children) |  |  | 8.76 | 8.76 |
| 1032. Malaria treatment (children 0-4) |  |  | 5.12 |  |
| 1033. DPT vaccination | 2.07 | 2.07 | 2.26 | 2.26 |
| 1034. Polio vaccine | 0.71 | 0.71 | 0.84 | 0.84 |
| 1035. Cotrimoxazole for children |  |  | 168.83 | 168.83 |
| 1036. Pediatric ART |  |  | 1,867.56 | 1,867.56 |
| 1045. Rotavirus vaccine | 6.88 | 6.88 | 6.91 | 6.91 |
| 1049. BCG vaccine | 0.17 | 0.17 | 0.20 | 0.20 |
| 1062. Parenteral administration of uterotonics |  |  | 5.59 | 5.59 |
| 1070. Newborn sepsis - Injectable antibiotics |  |  | 2.54 | 39.01 |
| 1071. Management of severe malnutrition (children) |  |  | 31.12 | 31.12 |
| 1072. Calcium supplementation for prevention and treatment of pre-eclampsia and eclampsia |  |  | 52.47 |  |
| 1073. Safe abortion |  |  | 24.54 | 24.54 |
| 1081. Management of pre-eclampsia (Magnesium sulphate) |  |  |  | 90.97 |
| 1082. Hypertensive disorder case management |  |  |  | 0.94 |
| 1083. Parenteral administration of anti-convulsants |  |  | 108.34 | 108.34 |
| 1094. Induction of labor for pregnancies lasting 41+ weeks |  |  |  | 11.58 |
| 1095. Pill - Standard daily regimen |  | 7.48 | 8.68 | 8.30 |
| 1096. Condom - Male |  | 7.79 | 8.18 |  |
| 1097. Injectable - 3 month (Depo Provera) |  | 4.31 | 5.08 |  |
| 1098. IUD - Copper-T 380-A IUD (10 years) |  |  | 2.03 | 1.87 |
| 1099. Implant - Jadelle (5 years) |  |  | 8.10 | 8.10 |
| 1100. Female sterilization |  |  |  | 6.46 |
| 1106. Antenatal Care (ANC): at least four visit |  |  | 2.45 | 2.45 |
| 1107. Management of other pregnancy complications |  |  | 70.04 | 70.04 |
| 1108. Deworming (pregnant women) |  |  | 0.10 | 0.10 |
| 1109. Pre-referral management of labor complications |  |  | 38.61 | 38.61 |
| 1110. Treatment of local infections (Newborn) |  |  | 6.40 | 97.53 |
| 1111. Mastitis |  |  | 29.62 | 29.62 |
| 1112. Treatment of postpartum hemorrhage |  |  | 59.09 | 59.50 |
| 1117. Treatment of PID (Pelvic Inflammatory Disease) |  |  | 5.58 | 5.58 |
| 1118. Treatment of urinary tract infection (UTI) |  |  | 22.56 | 22.56 |
| 1119. Cervical cancer screening |  |  | 11.39 | 11.39 |
| 1121. Identification and management of infertility |  |  |  | 2.83 |
| 1122. Treatment of severe diarrhea |  |  |  | 5.79 |
| 1123. Treatment of severe pneumonia |  |  |  | 73.01 |
| 1124. Treatment of severe malaria (children 0-4) |  |  |  | 59.04 |
| 1125. Treatment of severe measles |  |  |  | 160.61 |
| 1126. Malaria treatment (adults, excluding pregnant women) | 22.12 | 22.19 | 22.20 | 22.23 |
| 1128. First-line TB treatment: Initial treatment | 16.58 |  | 40.82 |  |
| 1129. First-line TB treatment: Previously treated | 16.58 |  | 40.82 |  |
| 1130. First-line TB treatment: Initial treatment for children | 16.58 |  | 64.86 |  |
| 1131. MDR-TB treatment |  |  | 1,798.34 | 1,798.34 |
| 1132. HPV vaccine | 9.19 | 9.11 | 9.31 | 9.31 |
| 1136. Indoor residual spraying | 9.70 |  |  |  |
| 1137. Food fortification |  |  |  |  |
| 1140. Condoms | 2.65 | 2.65 | 2.65 | 2.65 |
| 1141. Male circumcision |  |  | 16.31 | 16.31 |
| 1145. Youth focused interventions - Out-of-school |  |  |  |  |
| 1147. Interventions focused on female sex workers |  | 0.55 |  |  |
| 1151. Post-exposure prophylaxis |  |  | 4.66 | 4.66 |
| 1155. ART (Second-Line Treatment) for adults |  |  | 915.14 | 915.14 |
| 1156. Additional ART for TB patients |  |  |  |  |
| 1157. Diagnostics/lab costs for HIV+ in care VL test, CD4, Chemistry, hematology |  |  | 57.59 | 57.59 |
| 1158. Management of opportunistic infections associated with HIV/AIDS |  |  | 29.87 | 29.87 |
| 1159. Nutrition supplements |  |  | 147.25 | 147.25 |
| 1160. Screen HIV+ cases for TB |  |  |  |  |
| 1161. TB prevention: therapy for HIV+ |  |  | 10.98 | 10.98 |
| 1163. HIV prevention for TB patients |  | 1.00 | 1.00 | 1.00 |
| 1165. ART (+CPT) for TB HIV+ patients |  |  | 1.43 | 1.43 |
| 1167. Hep B vaccine to prevent liver cancer | 22.42 | 22.42 | 22.52 | 22.52 |
| 1170. Post-abortion case management |  |  | 60.97 | 60.97 |
| 1171. Ectopic case management |  |  |  | 40.75 |
| 1172. Treatment of malaria (pregnant women) |  |  | 9.74 | 9.74 |
| 1173. Maternal sepsis case management |  |  | 71.32 | 71.32 |
| 1175. Pentavalent vaccine |  |  | 2.53 |  |
| 1182. Daily iron supplementation for children 6 to 23 months (where anemia is >= 40%) | 2.28 |  | 2.41 |  |
| 1183. Feeding counselling and support for infants and young children in emergency situations | 0.69 |  | 0.69 | 0.69 |
| 1184. Feeding counselling and support for low-birth-weight infants |  |  | 8.41 | 8.41 |
| 1185. Infant feeding counselling and support in the context of HIV |  |  | 0.55 | 0.95 |
| 1188. Care for adults with low BMI | 48.49 |  | 48.49 | 48.49 |
| 1189. Nutritional care and support (HIV+ pregnant and lactating women) | 5.12 |  | 5.12 | 5.12 |
| 1190. Nutritional care and support (HIV+ children) |  |  | 1.27 | 0.95 |
| 1235. Daily iron and folic acid supplementation (pregnant women) | 1.78 |  | 1.49 | 1.18 |
| 1237. Management of moderate acute malnutrition (children) | 42.54 |  |  |  |
| 1242. Daily FAF, postpartum, anemic women |  |  | 0.57 | 5.75 |
| 1243. Intermittent FAF, postpartum, non-anemic pregnant women |  |  |  |  |
| 1245. Iodine supplementation in children |  |  |  |  |
| 1248. Vitamin A supplementation for treatment of xerophthalmia in children |  |  | 0.40 | 0.40 |
| 1249. Intermittent iron supplementation in children | 1.33 |  | 0.30 |  |
| 1250. Deworming (children) |  |  | 0.12 |  |
| 1260. Psychosocial care for peri-natal depression |  | 2.85 |  |  |
| 1264. Basic psychosocial treatment, advice, and follow-up for bipolar disorder, plus mood-stabilizing medication |  |  | 41.14 | 59.38 |
| 1267. Intensive psychosocial intervention for bipolar disorder, plus mood-stabilizing medication |  |  | 78.83 | 241.61 |
| 1270. Basic psychosocial treatment, advice, and follow-up for developmental disorders |  |  | 3.57 | 3.57 |
| 1271. Intensive psychosocial intervention for developmental disorders |  |  | 6.34 | 9.52 |
| 1272. Basic psychosocial treatment, advice, and follow-up for behavioral disorders |  |  | 3.57 | 3.57 |
| 1273. Intensive psychosocial intervention for behavioral disorders |  |  | 7.14 | 8.33 |
| 1275. Assessment, diagnosis, advice, and follow-up for dementia |  |  | 1.19 | 1.19 |
| 1277. Identification and assessment of new cases of alcohol use/dependence |  |  | 0.16 | 0.16 |
| 1278. Brief interventions and follow-up for alcohol use/dependence |  |  | 1.90 | 3.36 |
| 1281. Identification and assessment of new cases of drug use/dependence |  |  | 0.08 | 0.08 |
| 1282. Brief interventions and follow-up for drug use/dependence |  |  | 1.90 | 1.90 |
| 1284. Management of non-opioid/other drug withdrawal |  |  |  | 1.05 |
| 1285. Assess and care for person with self-harm |  |  | 0.08 | 0.08 |
| 1286. Basic psychosocial treatment, advice, and follow-up for self-harm/suicide |  |  | 1.59 | 2.38 |
| 1288. Basic psychosocial treatment and anti-depressant medication of first episode moderate-severe cases |  |  | 17.76 | 26.99 |
| 1289. Intensive psychosocial treatment and anti-depressant medication of first episode moderate-severe cases |  |  | 17.95 | 22.45 |
| 1290. Basic psychosocial support and anti-psychotic medication |  |  | 19.14 | 72.81 |
| 1291. Intensive psychosocial support and anti-psychotic medication |  |  | 64.99 | 71.97 |
| 1292. Basic psychosocial support, advice, and follow-up, plus anti-epileptic medication |  |  | 7.86 | 7.66 |
| 1295. Monitoring with microscopy: Test to monitor first-line drug treatment, new bacteriologically confirmed cases |  |  | 0.73 | 0.73 |
| 1298. Monitoring with culture: Test to monitor second-line treatment for RR-/MDR-TB |  |  |  |  |
| 1301. Drugs susceptibility testing for second line TB drugs |  |  |  |  |
| 1303. RR-Diagnosis Xpert: Resistance testing for new smear positive cases |  | 0.62 | 0.62 | 11.83 |
| 1304. RR-Diagnosis Xpert: Resistance testing for previously treated |  | 0.62 | 0.62 | 11.83 |
| 1308. Resistance testing LPA: For first-line drugs, new smear positive sputum |  |  |  |  |
| 1312. XDR-TB treatment |  |  |  | 7,756.13 |
| 1313. Ancillary drugs for adverse events treatment |  |  | 200.07 | 200.07 |
| 1314. Palliative care for TB patients |  |  |  |  |
| 1315. Patient support for new cases |  |  |  |  |
| 1316. Patient support for MDR and XDR cases |  |  |  |  |
| 1317. TB prevention: therapy for household contacts |  |  | 6.29 | 6.29 |
| 1318. Malaria diagnosis (malarial fevers) | 0.42 | 0.51 | 0.45 | 0.68 |
| 1323. Monitoring with microscopy: Test to monitor first-line drug treatment, previously treated cases |  |  | 0.73 | 0.73 |
| 1324. Patient support for previously treated cases |  |  |  |  |
| 1326. Family psychoeducation (ADHD) |  |  | 0.63 | 0.63 |
| 1327. Family psychoeducation (conduct disorder) |  |  | 0.63 | 1.90 |
| 1329. Basic psychosocial treatment and anti-depressant medication for anxiety disorders (moderate-severe cases) |  |  | 11.11 | 13.10 |
| 1330. Intensive psychosocial treatment and anti-depressant medication for anxiety disorders (moderate-severe cases) |  |  | 14.38 | 11.78 |
| 1348. Intensive psychosocial treatment and anti-depressant medication of recurrent moderate-severe cases on an episodic basis |  |  | 17.79 | 18.36 |
| 1349. Intensive psychosocial treatment and anti-depressant medication of recurrent moderate-severe cases on a maintenance basis |  |  | 15.78 | 51.48 |
| 1355. Monitoring with microscopy: Test to monitor second-line treatment for RR-/MDR TB |  |  | 0.73 | 0.73 |
| 1361. Resistance testing LPA: For first-line drugs, culture positive |  |  |  |  |
| 1362. Resistance testing LPA: For first-line drugs, previously treated TB cases |  |  |  | 19.78 |
| 1370. Monitoring X-rays: Test to monitor treatment for MDR or RR-TB |  | 0.08 | 0.08 | 0.08 |
| 1371. First-line TB treatment: Previously treated for children | 22.56 |  | 64.86 |  |
| 1449. Injury care due to intimate partner violence: physical assault |  |  | 5.26 | 5.26 |
| 1450. Injury care due to intimate partner violence: sexual assault |  |  | 0.70 | 0.70 |
| 1451. Family planning (contraceptives) for those not in union or below 15 years of age | 0.01 | 0.01 | 0.01 | 0.01 |
| 1452. Integrated management of common conditions in primary care | 1.21 | 1.21 | 1.21 |  |
| 1455. Schistosomiasis (PC for school age children) | 0.33 | 0.04 |  |  |
| 1457. Soil-transmitted helminthiasis (PC) | 0.14 | 0.04 |  |  |
| 1458. Onchocerciasis (PC) | 0.21 |  |  |  |
| 1459. Lymphatic filariasis (PC) | 0.21 | 0.04 |  |  |
| 1460. Trachoma (PC) | 0.41 |  |  |  |
| 1462. Buruli ulcer |  |  |  |  |
| 1463. Cutaneous leishmaniasis |  |  |  | 265.66 |
| 1464. Visceral leishmaniasis |  |  |  | 244.45 |
| 1465. Human African trypanosomiasis |  |  |  |  |
| 1466. Leprosy |  |  |  | 107.87 |
| 1467. Echinococcosis |  |  |  |  |
| 1468. Chagas disease |  |  |  |  |
| 1469. Lymphatic filariasis: hydrocele surgery |  |  |  |  |
| 1470. Trichiasis |  |  | 11.82 | 11.82 |
| 1471. Lymphedema management |  |  | 1.74 | 150.74 |
| 1472. Yaws |  |  |  |  |
| 1475. Sandflies | 3.63 | 0.18 |  |  |
| 1477. Rabies vaccination (canines) | 0.06 | 0.06 |  |  |
| 1478. Rabies post exposure prophylaxis | 0.40 | 0.40 |  |  |
| 1479. Seasonal malaria chemoprophylaxis |  | 13.10 | 4.53 |  |
| 1480. Malaria treatment (children 5-14) |  | 18.34 | 18.40 | 18.39 |
| 1481. Treatment of severe malaria (5+) |  |  | 16.86 | 18.24 |
| 1490. Diagnosis microscopy |  |  | 0.73 | 0.73 |
| 1491. Diagnosis GeneXpert |  | 0.62 | 0.62 | 11.83 |
| 1492. Diagnosis X-rays |  | 0.08 | 0.08 | 1.73 |
| 1493. Diagnosis culture |  |  |  |  |
| 1494. Screening: Intensified case finding |  |  | 0.36 | 2.01 |
| 1495. Screening: Active case finding |  |  | 0.55 | 1.97 |
| 1503. Resistance testing with LPA: For second-line drugs | 0.39 |  | 0.39 |  |
| 1505. Parenteral administration of antibiotics |  |  | 24.25 | 20.51 |
| 1506. Labor and delivery management |  |  | 24.17 | 24.17 |
| 1507. Manual removal of placenta |  |  | 12.84 | 12.84 |
| 1508. Removal of retained products of conception |  |  | 3.27 | 3.27 |
| 1509. Management of obstructed labor |  |  |  | 91.10 |
| 1510. Blood transfusion |  |  | 10.24 | 10.24 |
| 10001. Testing for PMTCT (Testing for pregnant women) |  |  | 3.73 | 3.73 |
| 10002. ART for PMTCT |  |  | 6.94 | 6.78 |
| 10003. NVP + AZT prophylaxis for HEIs |  |  | 2.78 | 2.78 |
| 10004. EID by DBS for HEI |  |  | 20.63 | 20.63 |
| 10005. POC (Point of care testing) for EID using Gene Xpert machine |  |  |  |  |
| 10006. Cotrimoxazole for HEI |  |  | 13.47 | 13.47 |
| 10007. Confirmatory test for HEI (ab test) |  |  | 3.73 | 3.73 |
| 10008. Cotrimoxazole for HIV positive women |  |  | 83.91 | 83.91 |
| 10009. IPT (INH preventive therapy) for HIV positive women |  |  | 1.24 | 1.24 |
| 10010. Testing for Partners of pregnant women |  |  | 3.73 | 3.73 |
| 10011. Screen HIV+ pregnant and lactating women for TB |  |  | 0.16 | 0.16 |
| 10012. Nutrition supplements for HIV positive pregnant, Lactating and HIV exposed Infants |  |  | 19.86 | 19.86 |
| 10014. Treatment of Syphilis in Pregnancy |  |  | 6.73 | 6.73 |
| 10016. Viral load test for HIV positive pregnant and lactating women |  |  | 58.22 | 58.22 |
| 10017. CD4 test for HIV positive pregnant and lactating women |  |  | 1.21 | 1.21 |
| 10018. Exclusive breast feeding |  |  | 0.40 |  |
| 10020. PrEP | 52.26 |  | 52.26 | 52.26 |
| 10021. Post GBV care |  |  | 6.47 |  |
| 10022. Voluntary counseling and testing |  |  |  |  |
| 10023. Targeted PITC |  |  | 4.10 | 4.10 |
| 10024. HIV self-testing |  |  |  |  |
| 10028. Cotrimoxazole for adults |  |  | 168.74 | 168.74 |
| 10029. Routine TB screening for PLHIV |  |  | 1.90 | 1.90 |
| 10030. Routine Screening of STI for PLHIV |  |  | 3.51 |  |
| 10031. Palliative care |  |  | 3.34 | 3.34 |
| 10032. Integration of family planning |  |  | 0.63 | 0.63 |
| 10037. Systematic screening |  |  | 0.68 | 7.74 |
| 10038. Hepatitis B and C viral load/PCR test |  |  |  | 15.96 |
| 10039. Anti-viral treatment for hepatitis B |  |  |  | 29.64 |
| 10040. Anti-viral treatment for hepatitis C |  |  |  | 264.22 |
| 10083. Case investigation - notification | 0.10 |  |  |  |
| 10084. Case investigation - focal test and treatment | 23.47 |  |  |  |
| 10085. Focus investigation - mapping (delinating) foci |  |  |  |  |
| 10086. Focus investigation - entomological investigation |  |  |  |  |
| 10087. Response to focus - vector control | 5.54 |  |  |  |
| 10088. Response to focus - mass drug administration | 24.16 |  |  |  |
| 10092. Screening and Management of STI |  |  | 2.92 | 2.92 |
| 10124. Scabies | 0.58 |  | 0.88 | 0.26 |
| 10125. Snake bite |  |  | 8.13 |  |
| 10129. Early ANC <16 weeks |  | 0.91 | 0.91 | 0.91 |
| 10130. Antenatal Care - First visit |  | 12.70 | 12.70 | 12.70 |
| 10132. Hepatitis (B and C) testing and treatment (pregnant women) |  |  | 0.45 | 0.45 |
| 10133. Cesarean section |  |  | 111.93 | 111.93 |
| 10134. 24-hour stay PNC |  |  | 1.80 | 1.80 |
| 10136. Management of uterine prolapse |  |  | 23.25 |  |
| 10137. Health education and counseling services | 0.31 | 0.31 | 0.31 | 0.31 |
| 10138. Contraceptive services | 0.71 | 0.71 | 0.71 | 0.71 |
| 10139. Oral contraceptives | 5.83 | 5.83 | 5.83 | 5.83 |
| 10140. Injectables | 2.06 | 2.06 | 2.06 | 2.06 |
| 10141. Implants | 10.56 | 10.56 | 10.56 | 10.56 |
| 10142. IUCD | 0.97 | 0.97 | 0.97 | 0.97 |
| 10143. Removal of IUCD methods | 0.10 | 0.10 | 0.10 | 0.10 |
| 10144. Removal of implant methods | 2.11 | 2.11 | 2.11 | 2.11 |
| 10152. HIV counseling and testing | 1.16 | 1.16 | 1.16 |  |
| 10155. GBV treatment |  |  | 0.69 | 0.69 |
| 10161. Treatment of fistula care |  |  | 0.45 | 0.45 |
| 10162. Provide Iron tablet service | 1.40 | 1.40 | 1.40 |  |
| 10163. Provide deworming among Adolescent girls | 0.16 | 0.16 | 0.16 |  |
| 10165. treatment of depression |  |  |  |  |
| 10167. Prevention management of FGM / Cutting/ |  |  | 0.67 | 0.67 |
| 10168. Health education on Self-care | 0.77 |  | 0.77 | 0.77 |
| 10169. Menarche and Menstrual Management | 1.93 | 1.93 | 1.93 |  |
| 10170. Screening & treatment of mental health problem in pregnant and postpartum women |  |  | 0.32 | 0.32 |
| 10171. Prevention of maternal alcohol use during pregnancy |  |  | 0.32 | 0.32 |
| 10172. Indicative screening for those with risk factor for anxiety and depression |  |  | 0.16 |  |
| 10178. Screening and proactive case finding of psychosis |  |  | 0.16 |  |
| 10182. Interventions to support caregivers of patients with dementia |  |  | 1.59 |  |
| 10188. Screening with TQ or the ACCESS portfolio |  |  | 0.16 |  |
| 10203. Acuity based triage service (facility level, community level mass casualty incident) |  |  |  |  |
| 10208. Trans vesical prostatectomy (TVP), Cysto-lithotomy |  |  |  | 91.69 |
| 10209. Rectal tube deflation for sigmoid volvulus |  |  | 7.28 | 7.28 |
| 10212. Cholecystectomy, cholecystostomy, CBD exploration, biliary bypass procedures and T-tube insertion for hepato-biliary pathologies |  |  |  | 71.13 |
| 10213. Repair of all types of hernias |  |  |  | 47.77 |
| 10214. Constructing and reversal of colostomies, colonic resection and anastomosis, Hemorrhoidectomies |  |  |  | 58.62 |
| 10215. Fistulotomies, fissure surgery, drainage of perianal abscesses and Hemorrhoidectomies |  |  |  | 28.11 |
| 10217. Gastric and esophageal resection for cancers and perforation |  |  |  |  |
| 10219. Management of Osteomyelitis |  |  |  | 74.18 |
| 10225. Extraction of Primary and Permanent tooth |  |  |  | 4.13 |
| 10226. Incision and drainage (periodontal and dental abscess) |  |  |  | 15.56 |
| 10227. Dental caries |  |  |  | 10.03 |
| 10228. Management of Maxillofacial and Dental Injuries |  |  |  | 36.26 |
| 10239. Dermatological curetting and electro surger |  |  |  | 12.40 |
| 10240. Cryotherapy |  |  |  | 0.83 |
| 10241. Laser therapy |  |  |  | 10.68 |
| 10252. Ear irrigation |  |  | 5.93 | 5.93 |
| 10255. Tonsillectomy |  |  |  | 36.04 |
| 10270. Management of Anemia |  |  | 76.52 | 76.52 |
| 10275. physiotherapy interventions |  |  |  | 36.87 |
| 10284. Urban Health extension program/implementing Family health team approach |  | 20.23 |  |  |
| 10286. Mobile Health team for pastoralist/semi-pastoralist and equity woredas |  | 8,740.28 |  |  |
| 10287. School and other institutional health |  |  |  |  |
| 10290. Emergency obstetric and surgical care at the health center |  |  | 920.41 |  |
| 10303. MCV (Measles) 2 | 0.49 | 0.49 | 0.59 | 0.59 |
| 10306. Hep birth dose | 7.54 | 7.54 | 7.64 | 7.64 |
| 10307. MR | 0.46 | 0.46 | 0.56 | 0.56 |
| 10308. Men A | 0.46 | 0.46 | 0.56 | 0.56 |
| 10309. Yellow fever | 1.04 | 1.04 | 1.08 | 1.08 |
| 10310. measles | 1.42 | 1.42 |  |  |
| 10311. Polio | 0.43 |  |  |  |
| 10313. Diagnosis with smear microscopy: Passive case finding |  |  | 0.72 |  |
| 10314. Diagnosis with smear microscopy: Contact investigation |  |  | 0.72 |  |
| 10315. Diagnosis with smear microscopy: House to house screening |  |  | 0.72 |  |
| 10316. Clinical diagnosis: Passive case finding |  |  |  |  |
| 10319. Leprosy treatment for MB adult |  |  | 1.90 |  |
| 10320. Leprosy treatment for MB Child |  |  | 1.90 |  |
| 10321. Leprosy treatment for PC adult |  |  | 1.90 |  |
| 10322. Leprosy treatment for PC child |  |  | 1.90 |  |
| 10323. Leprosy prevention of disability |  |  | 0.63 |  |
| 10324. Supplement deworming tablet in second trimester |  |  | 4.71 |  |
| 10416. Podoconiosis |  |  | 255.46 |  |
| 10418. Preconception care |  |  | 3.63 |  |
| 10419. Screening and management of GDM |  |  | 4.06 |  |
| 10420. Obstetric fistula |  |  |  |  |
| 10435. Advanced neonatal care |  |  |  | 122.64 |
| 10463. Proactive case finding for condut disorder |  |  |  |  |
| 10486. Periodontal Diseases |  |  |  | 81.16 |
| 10488. Management of Urinary Tract Infections (Lower) with antibiotics |  |  | 7.08 | 18.56 |
| 10493. Management of Glomerular diseases |  |  |  | 75.76 |
| 10495. medical management of Peptic Ulcer Diseases |  |  | 26.27 | 26.27 |
| 10496. Pneumonia |  |  | 16.75 | 16.75 |
| 10502. Dermatitis (Atopic dermatitis, Contact and seborrheic dermatitis) |  |  | 64.35 | 64.35 |
| 10503. Bacterial skin infections |  |  | 49.78 | 50.13 |
| 10504. Fungal skin diseases |  |  | 65.11 | 65.11 |

**Table S4. List of services from HSTP II costing with updated population in need estimates and sources**

|  | **Service** | **OHT value** | **New value** | **Source for new value** |
| --- | --- | --- | --- | --- |
| 1 | Care for adults with low body mass index | 0.12 | 0.16 | Tusa BS, Weldesenbet AB, Kebede SA. Spatial distribution and associated factors of underweight in Ethiopia: An analysis of Ethiopian demographic and health survey, 2016. PLoS One. 2020 Dec 1;15(12): e0242744. Doi: 10.1371/journal.pone.0242744 |
| 2 | Bacterial skin infections | 0.05 | 0.17 | IHME. GBD, 2019. Ethiopia. Bacterial skin diseases, incidence rate, all ages, both sexes, midpoint value. |
| 3 | Malaria treatment (children 5-14) | 0.20 | 1.00 | 100% of malaria cases require treatment. |
| 4 | Insecticide treated materials | 0.60 | 0.52 | Ethiopia Malaria Elimination Strategic Plan: 2021-2025. Ministry of Health. URI: <http://repository>.iifphc.org/xmlui/handle/123456789/1526. Date: 2020-08. |
| 5 | Dental caries | 0.05 | 0.64 | IHME. GBD, 2019. Ethiopia. Dental caribes of permanent and deciduous teeth, incidence rate, all ages, both sexes, midpoint value. |
| 6 | Condoms | 0.13 | 0.43 | Muche AA, Kassa GM, Berhe AK, Fekadu GA. Prevalence and determinants of risky sexual practice in Ethiopia: Systematic review and Meta-analysis. Reproductive Health. 2017 Sep;14(1):113. DOI: 10.1186/s12978-017-0376-4 |
| 7 | Dermatitis | 0.04 | 0.05 | IHME. GBD, 2019. Ethiopia. Dermatitis, incidence rate, all ages, both sexes, midpoint value. |
| 8 | Treatment of severe malaria (5+) | 0.01 | 1.00 | 100% of malaria cases require treatment. |
| 9 | Oral rehydration solution | 0.88 | 1.87 | IHME. GBD, 2019. Ethiopia. Diarrheal diseases, incidence rate, <5 years, both sexes, midpoint value. |
| 10 | Management of other pregnancy complications | 0.15 | 0.17 | IHME. GBD, 2019. Ethiopia. Maternal disorders, incidence rate, 15-49 years, females, midpoint value. |
| 11 | Repair of hernias | 0.07 | 0.065 | Abera, N. 2019. Prevalence of inguinal hernia and its risk factors in adult males admitted to surgical wards of teaching hospitals, in Addis Ababa, Ethiopia. |
| 12 | Periodontal diseases | 0.05 | 0.01 | IHME. GBD, 2019. Ethiopia. Periodontal diseases, incidence rate, all ages, both sexes, midpoint value. |
| 13 | Pre-referral management of labor complications | 0.15 | 0.17 | IHME. GBD, 2019. Ethiopia. Maternal disorders, incidence rate, 15-49 years, females, midpoint value. |
| 14 | Advanced neonatal care | 0.13 | 0.23 | IHME. GBD, 2019. Ethiopia. Neonatal disorders, incidence rate, <1 year, both sexes, midpoint value. |
| 15 | Zinc (diarrhea treatment) | 1.00 | 1.87 | IHME. GBD, 2019. Ethiopia. Diarrheal diseases, incidence rate, <5 years, both sexes, midpoint value. |
| 16 | Scabies | 0.41 | 0.08 | IHME. GBD, 2019. Ethiopia. Scabies, incidence rate, all ages, both sexes, midpoint value. |
| 17 | Management of obstructed labor | 0.12 | 0.004 | IHME. GBD, 2019. Ethiopia. Obstructed labor and uterine rupture, incidence rate, 15-49 years, females, midpoint value. |
| 18 | Medical management of peptic ulcer diseases | 0.03 | 0.0005 | IHME. GBD, 2019. Ethiopia. Peptic ulcer diseases, incidence rate, all ages, both sexes, midpoint value. |
| 19 | Deworming | 0.29 | 0.174 | Corrected PIN for children ages 2-4 |
| 20 | Family planning (contraceptives) for adolescents | 0.23 | 0.046 | Corrected PIN for adolescents <15 years |
| 21 | Post gender-based violence care | 0.12 | 0.001 | IHME. GBD, 2019. Ethiopia. Interpersonal violence, incidence rate, 15-49 years, females, midpoint value. |

**Table S5. List of standard treatment protocols from HSTP II costing that were revised**

| **Service** | **Original input** | **Revised input** |
| --- | --- | --- |
| - Malaria treatment (adults, excluding pregnant women) - Malaria treatment (children 0-4) - Malaria treatment (children 5-14) - Treatment of severe malaria (5+) - Case investigation - focal test and treatment - Response to focus - mass drug administration | Unit cost of primaquine phosphate (7.5 mg tablet) – US$ 4.70 | Unit cost of primaquine phosphate (7.5 mg tablet) – US$ 0.47 |
| Care for adults with low BMI | Nurses (6 days/visits, 60 minutes each) | Nurses (6 days/visits, 30 minutes each) |
|  | Generalists/primary care doctors (6 days/visits, 45 minutes each) | Generalists/primary care doctors (6 days/visits, 15 minutes each) |
|  | Nursing aides (6 days/visits, 60 minutes each) | Nursing aides (6 days/visits, 30 minutes each) |
|  | 100% of patients receive therapeutic spread, sachet 92g/CAR-150 (1 unit, 5 times per day, during 35 days) | 80% of patients receive therapeutic spread, sachet 92g/CAR-150 (1 unit, 5 times per day, during 35 days) |
| - Pneumonia treatment (children) - Malaria treatment (children) - Treatment of severe pneumonia - Treatment of severe malaria (children 0-4) - Treatment of severe measles - Periodontal diseases | Unit cost of paracetamol - 120mg/5ml – Syrup, 60ml - US$ 6.4 | Unit cost of paracetamol - 120mg/5ml – Syrup, 60ml - US$ 1.4 |
| Urban health extension program/implementing Family health team approach | Clinical officers/surgical technicians (96 days/visits, 250 minutes each) | Clinical officers/surgical technicians (2.5 days/visits, 50 minutes each) |
|  | Community health workers (96 days/visits, 250 minutes each) | Community health workers (2.5 days/visits, 50 minutes each) |
|  | Generalists/primary care doctors (96 days/visits, 250 minutes each) | Generalists/primary care doctors (2.5 days/visits, 50 minutes each) |
|  | Nurses (96 days/visits, 250 minutes each) | Nurses (2.5 days/visits, 50 minutes each) |
